# Segmental Dilatation of Intestine Presenting as Partial Intestinal Obstruction in a Child

**Published:** 2014-05-21

**Authors:** Rachid Khemakhem, Muhammad Riazulhaq, Elbager Othman Elhassan

**Affiliations:** Department of Pediatric Surgery, KFH, Taif, Saudi Arabia

**Keywords:** Intestinal obstruction, Intestinal dilatation, Segmental dilatation

## Abstract

Segmental dilatation of the intestine in pediatric age group is a rare entity. Patients usually present with partial intestinal obstruction which may delay surgical decision. Our case was an 18-month-old girl, who presented with partial intestinal obstruction, provisionally diagnosed as a case of Hirschsprung’s disease. Diagnostic evaluation with contrast study gave a clue of small intestinal obstruction with a dilated segment.

## INTRODUCTION

Segmental dilatation of the intestine (SDI) is a condition characterized by a sharply defined and markedly dilated segment of the intestine flanked by normal-caliber afferent and efferent bowels. Although segmental dilatation can involve the gastrointestinal tract anywhere from duodenum to distal colon, ileum is the most commonly affected site.[1] It is a rare cause of intestinal obstruction in neonates and children with a few cases described in the literature.[2] We report a case of segmental dilatation of ileum in a child.

## CASE REPORT

An 18-month-old girl presented with abdominal distension, bilious vomiting and failure to pass stool for 2 days. There was a history of off and on abdominal distention and episodes of vomiting with spontaneous resolution since birth. There was no history of delayed passage of meconium or failure to thrive. On examination, abdomen was hugely distended, soft, and with no tenderness. Per rectal examination was unremarkable. Abdominal x-ray showed a big air fluid level occupying the entire abdomen associated with multiple other air fluid levels. Blood investigations were normal. A nasogastric tube was inserted and IV fluids were started. The patient was kept under observation. She passed stool occasionally but abdomen remained distended with persistent bilious aspirate. Repeat radiograph showed same findings. Contrast enema showed a non distended colon localized in the left part of the abdomen with a hugely distended loop occupying the right flank and iliac fossa (Fig.1).

**Figure:**
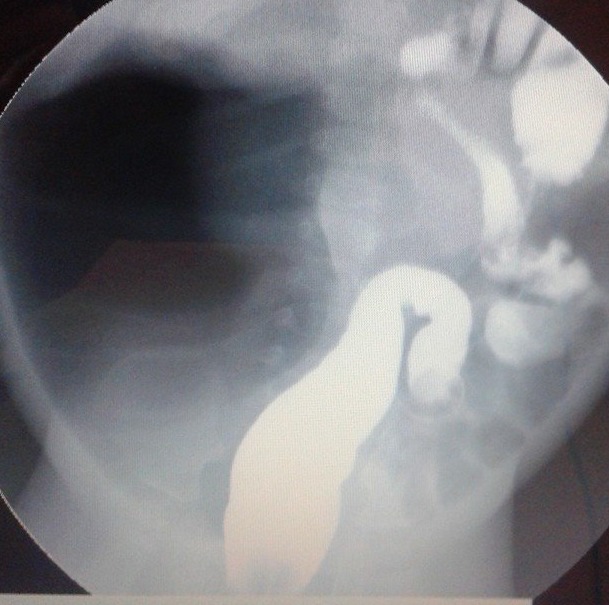
Figure 1: Contrast study showing massively dilated bowel loop in the right side of the abdomen with normal colon displaced to the left side.

Exploratory laparotomy showed a segmental dilatation of small bowl, 70cm proximal to ileocecal junction, which was about 13 cm x 10 cm in size, and the affected section of the intestine was dilated regionally to six times the normal value in diverticular fashion (Fig. 2). The dilated segment was in continuity with normal caliber distal bowel and mildly dilated proximal bowel and was twisted 90 degrees but viable. There was associated malrotation present. The distended loop was resected and ileo-ileal anastomosis done. Ladd’s procedure was also added. Postoperatively, the patient did well. Histopathology showed chronic inflammation of both mucosa and submucosa, with superficial ulceration without evidence of abnormal heterotopic gastric mucosa and with the presence of normal ganglion cells. The resected appendix was normal.

**Figure:**
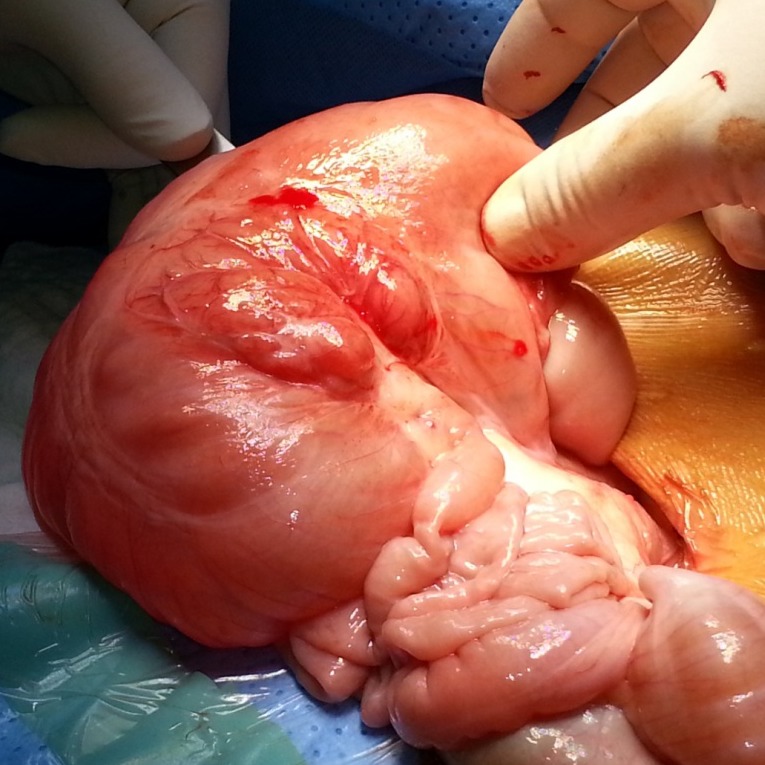
Figure 2: Operative photograph showing sharply demarcated segmental dilatation of the small bowel in continuity with the normal intestine.

## DISCUSSION

SDI is an uncommon entity described for the first time in the colon by Swenson and Rathauser in 1959. SDI can affect colon, ileum, and jejunum.[3]

Criteria for diagnosis of this entity, as proposed by Swenson and Rathauser are 

(1) Limited bowel dilatation with 3- to 4-fold increased size.

(2) Abrupt transition between dilated and normal bowel.

(3) No intrinsic or extrinsic barrier distal to the dilatation.

(4) Clinical picture of intestinal occlusion or sub-occlusion.

(5) Normality of neuronal plexus, and

(6) Complete recovery after resection of the affected segment.

SDI can present as an isolated entity or may be associated with other congenital malformations.[2,4] These associated malformations are found in more than 50% of the cases. Digestive malformations are essentially represented by disorders of rotation and fixation, as in our case where malrotation was found. Omphalocele is presumed to play a role in its etiogenesis.[2]

Clinical presentation depends upon site of SDI and age of presentation. More than 50% of the SDIs occur in neonatal period. The diagnosis is most frequently made at surgical intervention. Equally, the SDI can be possibly revealed through a complication: mainly a bowel obstruction. Cases involving the colon have a clinical picture very similar to that of Hirschsprung’s disease.[2,6] Beyond the neonatal period, the SDI can present with chronic abdominal pain, distention and anemia, which would follow the ulceration of the dilated loop.[2,7]

Radiologic findings are not specific and abdominal x-ray shows signs of intestinal obstruction with one big fixed distended loop with hydroaeric level. A contrast x-ray study can identify the dilated segment. Computed tomography may show a large saccular dilatation of a bowel loop, containing a mixture of oral contrast material and feces. The usual finding on laparotomy is a localized dilation of an isolated, well defined segment of bowel with apparently normal bowel proximal and distal to this segment. The obstruction in these cases is functional and non-mechanical because the lumen of the dilated segment is continuous with rest of the intestine as in our case.[6] In conclusion, SDI should be included among the differential diagnoses of intestinal obstruction in neonates and children especially if there is a hugely distended loop on abdominal x-ray.

## Footnotes

**Source of Support:** Nil

**Conflict of Interest:** None declared

